# Excess Mortality as the Primary Mediator of COVID-19’s Impact on Life Expectancy in Europe: A Multilevel Longitudinal Analysis of Regional Disparities

**DOI:** 10.3390/medicina62061020

**Published:** 2026-05-25

**Authors:** Viorel Țarcă, Solange Tamara Roșu, Elena Cojocaru, Lăcrămioara Ionela Butnariu, Iulia Cristina Roca, Ancuța Lupu, Dana Elena Mindru, Paula Popovici, Elena Țarcă

**Affiliations:** 1 Faculty of Medicine, Apollonia University, Strada Păcurari nr. 11, 700511 Iasi, Romania; viorel.tarca@univapollonia.ro; 2 Grigore T. Popa University of Medicine and Pharmacy, 700115 Iasi, Romania; elena2.cojocaru@umfiasi.ro (E.C.); ionela.butnariu@umfiasi.ro (L.I.B.); iulia.roca@umfiasi.ro (I.C.R.); ancuta.ignat1@umfiasi.ro (A.L.); mindru.dana@umfiasi.ro (D.E.M.); paula.popovici@umfiasi.ro (P.P.); tarca.elena@umfiasi.ro (E.Ț.)

**Keywords:** COVID-19, life expectancy, excess mortality, health disparities, multilevel modeling, Europe, vaccination, health expenditures

## Abstract

*Background and Objectives:* The COVID-19 pandemic caused unprecedented mortality shocks worldwide, but its differential impact across European regions and the mediating mechanisms remain inadequately quantified. *Materials and Methods:* We conducted a longitudinal multilevel analysis using data from 29 European countries (2015–2023; *N* = 261 country-years). Linear mixed models estimated the impact of the pandemic on life expectancy, controlling for regional differences, vaccination rates, healthcare expenditures, gross domestic product, and excess mortality. The primary outcome was national life expectancy at birth. *Results:* The pandemic period was associated with an average reduction of 1.12 years in life expectancy (95% CI: 0.95 to 1.49, *p* < 0.001) after adjusting for pre-existing trends. Eastern Europe experienced 56% greater impact than Western Europe (interaction β = −0.623, *p* = 0.002). Excess mortality emerged as the primary mediator, explaining 79% of the pandemic effect. Each 1% increase in excess mortality reduced life expectancy by 0.091 years (*p* < 0.001). Healthcare expenditures showed protective effects (β = 0.000327 per purchasing power standards (PPS), *p* = 0.049), while vaccination rates, as a direct predictor, were not significantly associated with life expectancy in multivariate models. This critical finding on vaccination rates does not imply biological inefficacy but rather suggests a misspecification of its role. *Conclusions:* Excess mortality, rather than its direct component, COVID-19-specific mortality, appears to mediate most of the pandemic’s impact on life expectancy. Regional disparities reflect structural differences in healthcare systems and socioeconomic conditions more than differential vaccination uptake. The protective effect of vaccination on life expectancy operates entirely through the reduction in excess mortality. Consequently, health policies should prioritize strengthening resilient health systems as well as disease-specific interventions.

## 1. Introduction

The COVID-19 pandemic represents the most severe global health crisis in a century, with profound implications for population health indicators. Globally, COVID-19 is thought to have claimed over 1.8 million deaths only in 2020 [1], and the WHO has estimated that 14.9 million excess deaths from COVID-19 occurred globally in 2020–2021 [2]. Europe, despite its advanced healthcare systems, experienced significant mortality shocks, with reported COVID-19 deaths exceeding 2 million by 2023. However, the pandemic’s true impact extends beyond direct COVID-19 mortality to include excess deaths from disrupted healthcare services, delayed treatments, and socioeconomic consequences [3]. Life expectancy at birth serves as a comprehensive indicator of population health, sensitive to mortality shocks across all age groups [4]. Between 2019 and 2021, the global life expectancy at birth decreased by 1.6 years (1.0–2.2), reversing historical trends [5]. Moreover, population aging is rapidly increasing worldwide, with projections indicating that by 2030, one in six people will be over the age of 60. This shift is already visible in Europe, where gains in life expectancy have slowed, the gap between years lived in good health and total lifespan is widening, and labor shortages are becoming more pronounced [6]. Previous studies have documented temporary declines in life expectancy during the pandemic [1,7], but several critical questions remain unanswered. First, the magnitude of regional disparities within Europe requires quantification, particularly the East–West divide that characterizes many health outcomes [8,9,10]. Second, the relative importance of different protective factors—including vaccination, healthcare capacity, and economic resources—remains contested. Third, the mediating role of excess mortality versus COVID-19-specific mortality needs clarification.

This study addresses these gaps through a multilevel longitudinal analysis of 29 European countries from 2015 to 2023. We aim to (1) quantify the pandemic’s impact on life expectancy, adjusting for pre-existing trends; (2) examine regional variations in vulnerability; (3) identify factors moderating the impact; and (4) test whether excess mortality mediates the relationship between the pandemic and life expectancy.

### Research Questions

The investigation is guided by the following questions:

1. What was the magnitude of the life expectancy decline attributable to the pandemic period in Europe?
2. Were there significant regional differences in the scale of this impact?
3. Which factors (healthcare expenditure per capita, gross domestic product (GDP) per capita, vaccination coverage) significantly moderated the pandemic’s effect on life expectancy?
4. Through what primary mechanism (e.g., direct mortality, healthcare system collapse) did the pandemic predominantly influence life expectancy?

## 2. Materials and Methods

### 2.1. Study Design and Data Sources

We conducted a longitudinal study using annual data from 2015 to 2023. Data were collected from multiple sources: life expectancy, GDP, healthcare expenditures, and demographic indicators from Eurostat; vaccination data from Our World in Data and the WHO; and excess mortality statistics from Eurostat’s monthly database [3,11,12]. The analysis included 29 European countries representing four regions: North (Denmark, Finland, Iceland, Norway, Sweden, Estonia, Latvia, Lithuania), South (Cyprus, Greece, Italy, Malta, Portugal, Spain), West (Austria, Belgium, France, Germany, Luxembourg, Netherlands), and East (Bulgaria, Croatia, Czechia, Hungary, Poland, Romania, Slovakia, Slovenia). This yielded 261 country-year observations (29 countries × 9 years).

### 2.2. Variables Included in the Study

The selection of variables for the multilevel model was guided by a comprehensive theoretical framework integrating demographic transition theory, social determinants of health, and health system resilience concepts. Each variable serves a distinct purpose in testing specific pathways through which the COVID-19 pandemic affected European life expectancy.

Outcome: Life expectancy at birth (years) represents the ultimate summary measure of population health, being a reliable indicator of the COVID-19 pandemic’s overall demographic impact because it is sensitive to mortality shocks across all age groups [12]. Life expectancy at birth is the average number of years a newborn is expected to live, assuming they experience current mortality rates throughout their lifetime.

We acknowledge the conceptual overlap between excess mortality and life expectancy, as the latter is derived from mortality schedules. Our analytical use of excess mortality as a predictor will therefore be best understood as a mediation or decomposition approach rather than a causal claim of independent exogeneity. The coefficient estimates the contribution of the mortality shock to the observed change in life expectancy, consistent with standard practices in demographic crisis research [1,13,14,15].

Primary Predictors:

➢ Pandemic period (the primary treatment variable in initial models): binary constructed variable (0 = pre-COVID-19 period 2015–2019; 1 = COVID-19 period 2020–2023). Represents exposure to the systemic shock of the pandemic crisis. Its initial coefficient captures the average discrete impact before introducing mediating mechanisms.
➢ Year: Continuous variable (2015–2023). Controls for the secular, pre-existing trend of technological, medical, and social progress that increases life expectancy annually. This variable is essential for isolating the net effect of the pandemic from ongoing improvements.
➢ Region: Categorical variable (1 = North; 2 = South; 3 = East; 4 = West) that captures deep-seated, structural heterogeneity. Represents clusters of countries with shared historical, institutional, economic, and epidemiological trajectories. Tests the hypothesis that pre-existing structural vulnerabilities determined crisis outcomes.

Covariates:

➢ GDP per capita (purchasing power standards—PPSs; EU27 from 2020—per capita): structural moderator (continuous). Indicates the resources available for public and private investment in health determinants (education, environment, infrastructure), as well as overall economic capability and development level. At the same time, it determines if wealthier countries had a stronger shock buffer [16].
➢ Healthcare expenditures (purchasing power standard of current health care expenditure per inhabitant): structural moderator (continuous). Measures specific investment in the health system, being a more direct measure of health system capacity and resilience than GDP. Tests the hypothesis that strong, well-funded systems can better absorb the surge, keep key services running, and so reduce life expectancy loss [17].
➢ Percentage of population with at least one vaccine dose (vaccination coverage): crisis response continuous variable. It serves as the main biomedical intervention during the pandemic. In the proposed model, we will examine its potential protective moderator role: was the impact on life expectancy less severe in places with higher coverage? [11]
➢ Percentage of population aged 65 years and over: controls for demographic aging (continuous). Older populations have higher baseline mortality and are more vulnerable to COVID-19 [18]
➢ Population density (inhabitants per square kilometer): controls for potential transmission dynamics. Denser populations may facilitate virus spread, potentially leading to higher infection rates and mortality [19]
➢ Excess mortality offers a comprehensive and robust measure of population-level mortality by capturing both direct and indirect deaths from all causes (from overloading the medical system, delay in the evaluation and treatment of chronic conditions, socio-economic disruptions), thereby enabling meaningful comparisons across countries and time periods without reliance on cause-specific reporting [20]. The excess mortality indicator compares each period’s total number of deaths from all causes to a historical baseline from prior years in a time frame unaffected by the COVID-19 epidemic. In the present case, the average number of deaths that took place between 2016 and 2019 serves as the baseline. The excess mortality index does not account for age class or sex, nor does it differentiate between the causes of death.

### 2.3. Statistical Analysis

We employed linear mixed models with random intercepts for countries to account for clustering. The analysis proceeded in five stages. Model fit was assessed using Akaike Information Criterion (AIC), Bayesian Information Criterion (BIC), and R^2^. Assumptions of normality, homoscedasticity, and independence were checked through residual analysis. Intraclass correlation coefficients (ICC) quantified between-country variance. All analyses were conducted in SPSS 27 using Restricted Maximum Likelihood (REML) estimation. Statistical significance was set at *p* < 0.05 (two-tailed).

## 3. Results

Our research begins with a descriptive summary of the fundamental variables for the 29 European countries over a nine-year observation period (2015–2023). The tables below show the central patterns and distribution of key demographic, economic, and health system indicators, laying the basis for the pandemic’s impact and illustrating the continent’s deep heterogeneity. Table 1 presents the descriptive statistics for the core variables across the 261 country-year observations comprising the analytical panel (2015–2023). The data reveal the fundamental parameters and underlying heterogeneity of the European demographic and socio-economic landscape during the study period.

The central outcome variable, life expectancy at birth, exhibits a general mean of 77.37 years (SD = 3.72). The considerable range, from a minimum of 68.0 years (Bulgaria, 2021) to a maximum of 81.8 years (Iceland, 2021), signifies profound and persistent cross-national disparities in population health, indicative of deep-seated structural inequalities.

Economic and health system capacity, measured by GDP per capita (PPS) and healthcare expenditure per capita (PPS), demonstrate significant dispersion. The mean GDP of 33,495 PPS (SD = 14,490) and the mean health expenditure of 2787 PPS (SD = 1134) are characterized by substantial standard deviations relative to their means, confirming a high degree of economic and fiscal heterogeneity. The near seven-fold difference between the minimum and maximum values for health expenditure underscores stark contrasts in resource allocation and health system investment priorities across nations.

The pandemic-specific variable, percentage of the population vaccinated with at least one dose, shows a mean of 21.48% with a very high standard deviation (31.78%). This distribution is intrinsically shaped by the temporal scope of the data, which includes pre-pandemic years (0% vaccination) and the subsequent rollout period, culminating in a maximum observed coverage of 94.28% in Portugal 92023). This variable thus captures the dynamic and uneven diffusion of the primary biomedical intervention across the continent. Demographic controls display differing patterns. The percentage of the population aged 65 and above averages 19.12% (SD = 2.62%), reflecting Europe’s uniformly advanced age-structural transition, albeit with moderate variation. In stark contrast, population density is characterized by extreme positive skewness (Mean = 171.5 inhabitants/km^2^, SD = 283.6), indicative of the coexistence of vast, sparsely populated territories and intensely urbanized agglomerations within the sample.

Finally, the key mechanistic variable, excess mortality, has a mean of +4.59% (SD = 7.23%) for the full period. The values range from −6.64% (Romania, 2023) to +38.01% (Bulgaria, 2021), quantifying the severe but highly variable mortality shock incurred during the pandemic years relative to the pre-pandemic (2016–2019) baseline. This metric encapsulates the combined direct and indirect mortality consequences of the crisis, serving as the proximate determinant of life expectancy trends.

Collectively, these statistics establish the baseline variance and central tendencies that inform the subsequent multilevel modeling, highlighting the significant between-country differences that the analytical framework is designed to parse. Before model estimation, a comparative descriptive analysis was conducted to characterize the fundamental differences between the pre-pandemic (2015–2019) and pandemic (2020–2023) periods. Table 2 presents the means and standard deviations of the core variables across these two distinct epochs.

This preliminary comparison serves to quantify the immediate contextual shifts—in economic conditions, healthcare investment, and mortality—against which the more nuanced, adjusted effect of the pandemic on life expectancy must be assessed. The observed stability in the aggregate life expectancy mean belies the significant changes in its underlying determinants, highlighting the necessity for the multivariate, longitudinal approach employed in the subsequent analysis.

The analysis begins by breaking down the life expectancy trend into four key regional categories: north, south, east, and west. This stratification demonstrates a disparate impact of the pandemic period across Europe’s major geographical and socioeconomic regions. While the Northern and Western regions had moderate stability or slight nominal advances, the Southern and, most notably, Eastern regions experienced significant declines (Table 3).

This early geographical split offers an essential framework for the strong interaction effects investigated in the later multilevel models, suggesting that the pandemic’s demographic impact was not a uniform continental phenomenon but rather regionally regulated. The profound regional disparities that characterized Europe before the pandemic are systematized in Table 4, which presents the pre-pandemic (2015–2019) averages for key structural variables.

This comparison analysis reveals the deep gradients that shape the continent’s vulnerability landscape. A substantial East–West divide is seen, with a pre-existing life expectancy gap of 5.82 years and an almost two-fold disparity in economic capacity (GDP PPS) and healthcare investment. These significant structural differences in resources and health outcomes created a distinct hierarchy of systemic resilience, giving critical context for evaluating the varying severity of the pandemic’s impact revealed by the longitudinal models. The Pearson correlation matrix (Table 5) elucidates the fundamental bivariate relationships structuring the European health landscape.

Life expectancy exhibits a strong, positive association with both GDP per capita (r = 0.560, *p* < 0.01) and, more robustly, with healthcare expenditures per capita (r = 0.705, *p* < 0.01), affirming the primacy of direct health investment. The vaccination variable shows significant but modest correlations with economic and health system capacity (r = 0.322 and 0.367, respectively, *p* < 0.01), indicating its rollout was intertwined with structural advantages. Notably, excess mortality demonstrates a weak and non-significant direct correlation with life expectancy at the bivariate level (r = −0.104, *p* > 0.05), a counterintuitive finding that underscores the necessity of a longitudinal, multivariable modeling approach to disentangle its effect from confounding temporal and regional trends.

The correlation matrix reveals a notable and statistically significant positive association between excess mortality and vaccination coverage (r = 0.497, *p* < 0.01). This counterintuitive finding is a classic example of ecological fallacy and reverse causality at the aggregate level. It does not imply that vaccination caused higher mortality; rather, it reflects the dominant temporal confounding within the panel data: countries implemented mass vaccination campaigns precisely during the periods of highest pandemic intensity (2021–2022), when excess mortality was also at its peak. Consequently, higher observed vaccination rates are ecologically linked to the acute crisis years. This spurious relationship underscores the critical limitation of bivariate correlations for inferring causal mechanisms in a longitudinal crisis context and justifies the necessity of the multilevel modeling framework, which controls for the temporal variable (Year) and isolates the net effect of each predictor.

**Figure 1:**
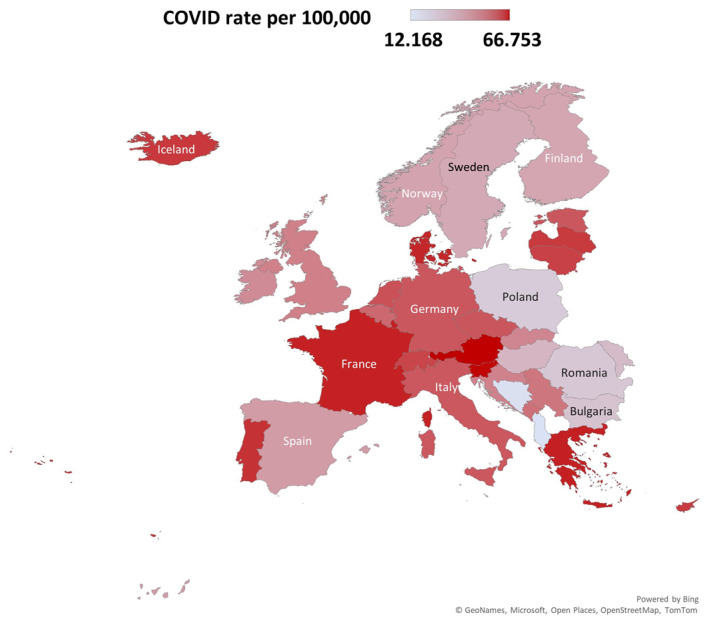
Cumulative COVID-19 incidence rate per 100,000 population (2020–2023).

**Figure 2:**
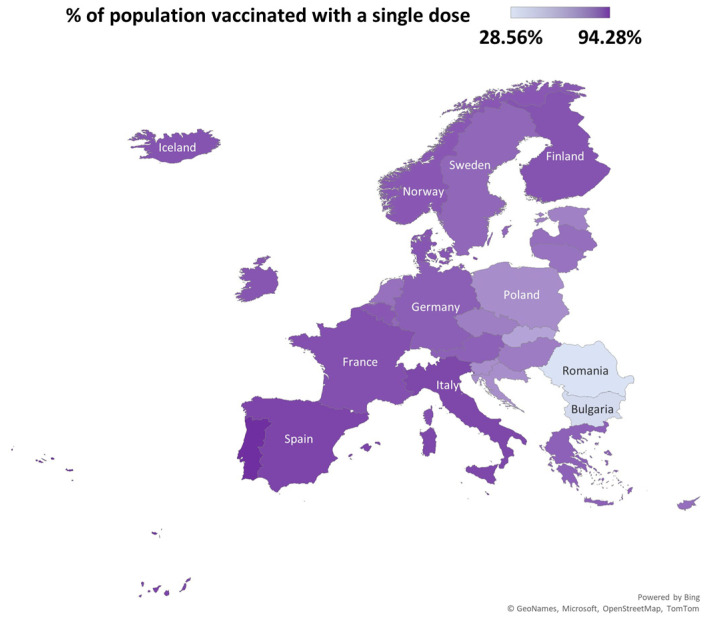
Vaccination coverage (% of population vaccinated with 1 dose, 2021–2023).

The aggregate statistics mask significant national-level variation in both the intensity of the pandemic and the scope of the public health response. This heterogeneity is illustrated in Figure 1 and Figure 2. The cumulative incidence of confirmed COVID-19 cases varied dramatically across Europe, ranging from 12,168 per 100,000 in Albania to 66,753 per 100,000 in Austria (Figure 1). This near 5.5-fold difference reflects not only genuine variations in viral transmission but also profound disparities in testing capacity, surveillance infrastructure, and national reporting protocols.

Similarly, the primary biomedical countermeasure, vaccination, achieved widely divergent levels of coverage. At the conclusion of the major rollout campaigns, coverage ranged from 28.56% in Romania to 94.28% in Portugal. This extreme range, spanning from hesitant adoption to near-universal coverage, highlights critical differences in public trust, logistical efficiency, and the political commitment to vaccination as a central pandemic mitigation strategy (Figure 2).

To elucidate the dynamic pathways underlying the aggregate results, the following series of figures presents the longitudinal evolution of core indicators—life expectancy, healthcare expenditure, GDP, and vaccination coverage—disaggregated by region from 2015 to 2023. This temporal disaggregation reveals not only the immediate shock of the pandemic in 2020–2021 but also the divergent trajectories of recovery and the persistence of pre-existing regional gradients. Analyzing these parallel trends is essential for moving beyond static comparisons and understanding how the crisis differentially interacted with the structural capacities and responses of each European bloc.

**Figure 3:**
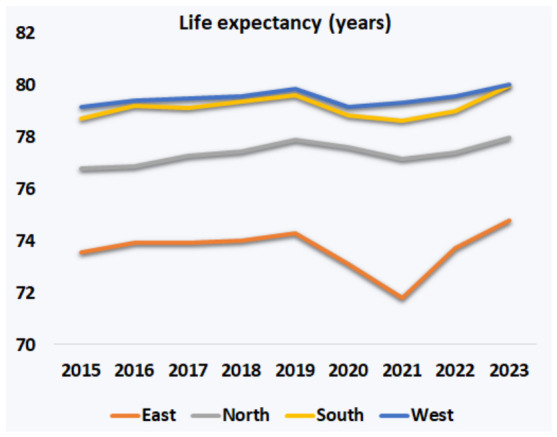
Longitudinal evolution of life expectancy by region (2015–2023).

Figure 3 reveals the distinct dynamics of life expectancy across Europe’s four regions before, during, and after the acute phase of the pandemic. A general upward trend is observable in the pre-pandemic period (2015–2019) across all regions, with consolidated annual gains. However, the impact of the pandemic in 2020 was immediate and pronounced, triggering a sharp decline or stagnation. The year 2021 represents the nadir for three of the regions, reflecting the peak lagged effect of mortality, with Eastern Europe recording the most drastic decline (from 74.30 in 2019 to 71.79 in 2021). Remarkably, except for the East, all regions appear to have reached or even exceeded their pre-pandemic levels by 2023, suggesting a demographic rebound. This trajectory viscerally highlights not only the magnitude of the shock but also the differing speed of recovery, with the East lagging in the recoupment of lost life expectancy.

**Figure 4:**
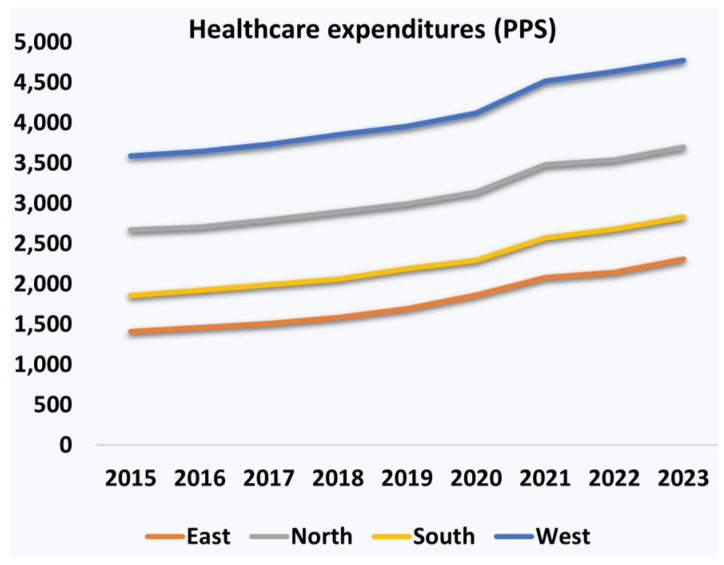
Longitudinal trajectory of healthcare expenditure per capita (PPS) by region (2015–2023).

The temporal evolution of healthcare investment (Figure 4) demonstrates a consistent, absolute increase across all European regions throughout the entire nine-year period, including during the pandemic.

The data reveal a significant and widening absolute gap in health spending between Western Europe and other regions, particularly the East. While all regions show an accelerated rate of increase from 2020 onwards—likely reflecting emergency COVID-19 allocations—the pre-existing hierarchical structure remains rigidly intact. The West not only maintained but expanded its substantial spending lead, underscoring that the pandemic crisis prompted a proportional, rather than redistributive, fiscal response in health financing across the continent. This sustained investment gradient is a critical structural factor underpinning the observed disparities in health system resilience and population health outcomes.

**Figure 5:**
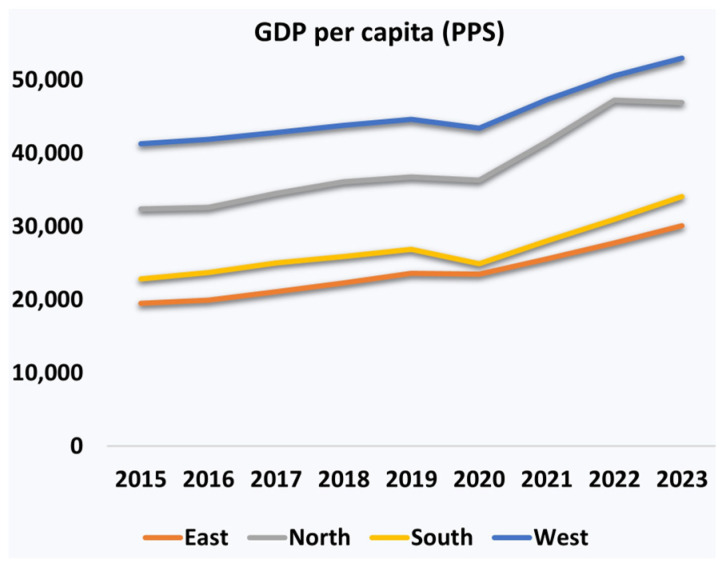
Longitudinal trajectory of gross domestic product per capita (PPS) by region (2015–2023).

The economic trajectory across European regions (Figure 5) illustrates both shared cyclical patterns and persistent structural divides. All regions exhibited steady pre-pandemic growth until 2019. The year 2020 marked a universal, though uneven, contraction linked to the initial economic shock of the pandemic, with Southern Europe experiencing the most pronounced decline. The subsequent rebound from 2021 onward was vigorous, leading to new economic peaks in all regions by 2023. However, this parallel recovery did not alter the fundamental economic hierarchy.

The proportional gap between the highest-spending (West) and lowest-spending (East) regions remained largely constant throughout the period, demonstrating that the pandemic-induced economic volatility occurred within a rigid framework of pre-existing and deeply entrenched regional inequalities in economic capacity.

**Figure 6:**
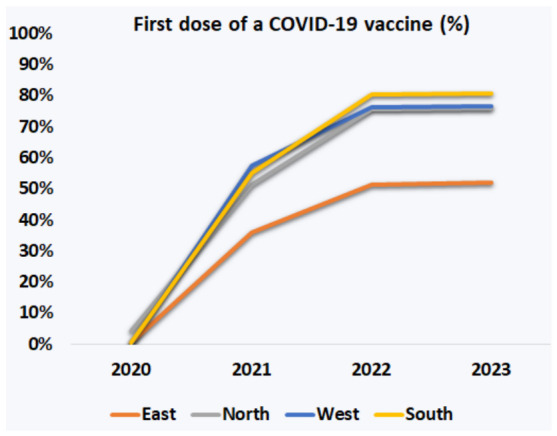
Vaccination rollout dynamics by region: cumulative coverage with at least one dose (%).

The phased rollout of COVID-19 vaccination (Figure 6) captures the rapid yet regionally divergent deployment of the primary biomedical intervention. Initial coverage by the end of 2020 was minimal across all regions.

The year 2021 witnessed a massive scale-up, with Western and Southern Europe achieving a significant lead, surpassing 50% coverage. By 2022, a clear plateau pattern emerged: Southern and Western Europe reached a high-coverage equilibrium (approximately 76–81%), while Northern Europe followed closely. Eastern Europe, however, consistently lagged, stabilizing at a markedly lower plateau of around 52% coverage. This trajectory illustrates not just a delay in the Eastern rollout but a fundamental disparity in final uptake, highlighting a critical divide in the implementation and public acceptance of the pandemic response that aligns with the region’s broader vulnerability pattern.

The observed descriptive patterns establish the necessary context but cannot adjudicate between competing explanations for the documented life expectancy losses. To quantify the net pandemic effect, test for regional effect modification, and evaluate the role of hypothesized protective factors, we implement a longitudinal multilevel modeling strategy. This inferential approach explicitly models the nested data structure, controls for confounding temporal trends, and enables the formal testing of interaction effects, thereby moving the analysis beyond associative patterns toward a causal understanding of the observed demographic changes.

To robustly assess the pandemic’s impact, we employed a hierarchical (multilevel) modeling strategy using the mixed procedure in SPSS 27 with Restricted Maximum Likelihood (REML) estimation. This approach is essential for our data structure, where repeated annual observations (Level 1: years) are nested within countries (Level 2). It allows us to partition variance, model within-country change over time, and account for the non-independence of observations from the same country.

The strategy was sequential and hypothesis-driven. We began with a simple null model and progressively added complexity, testing specific research questions at each stage. The improvement of each model was rigorously evaluated using information criteria (AIC, BIC), likelihood-ratio tests, and the proportion of explained variance (R^2^).

Table 6 below outlines the staged model-building process, its theoretical rationale, and the key statistical outcomes that guided our progression to the final, optimized model.

### 3.1. The Sequential Modeling (Table 6) Revealed a Clear Narrative

#### 3.1.1. Model 0 (Null Model)

The analysis commences with a null (empty) multilevel model to partition the total variance in life expectancy. The exceptionally high Intra-class Correlation Coefficient (ICC) of 97.87% reveals that nearly all observable variance resides between countries rather than within them over time. This finding provides the foundational justification for the hierarchical modeling approach, as it confirms that country-specific, time-invariant factors dominate the data structure. Ignoring this clustering would constitute a critical violation of independence assumptions, leading to biased standard errors. The model thus establishes a statistical baseline, quantifying the substantial heterogeneity that any subsequent explanatory model must account for.

#### 3.1.2. Model 1 (Time and Pandemic Effect)

Introducing fixed effects for the linear secular trend (Year) and the binary pandemic period (Pandemic_year), Model 1 quantifies the pandemic’s gross average impact. The results indicate a robust pre-pandemic annual increase in life expectancy of 0.254 years. Against this backdrop of progress, the pandemic period is associated with a significant net decline of 1.223 years. Crucially, this coefficient represents the unadjusted shock, suggesting the crisis erased approximately five years of prior gains. However, this model remains underspecified, as it applies an identical shock to all countries, disregarding their profound pre-existing structural differences, which were vividly illustrated in the descriptive analysis.

#### 3.1.3. Model 2 (Incorporating Regional Structure)

Model 2 advances the specification by introducing Region as a categorical fixed effect, acknowledging Europe’s entrenched geographic and socio-economic stratification. The results confirm a massive and significant pre-pandemic East–West life expectancy gap of 5.83 years, even after controlling for the temporal trend and pandemic shock. The notable improvement in model fit (ΔAIC = −20.75) statistically validates the region as a fundamental explanatory factor. Interestingly, the pandemic coefficient remains unchanged from Model 1, indicating that the regional variable captures baseline disparities but does not yet explain differential vulnerability to the shock—an assumption challenged by descriptive trends and addressed in the next step.

#### 3.1.4. Model 3 (Testing Differential Vulnerability via Interaction)

To test the core hypothesis of heterogeneous impact, Model 3 introduces an interaction between Region and Pandemic_year. This represents a major conceptual and statistical improvement (ΔAIC = −19.19), confirming that the pandemic’s effect was not uniform. The significant negative interaction for Eastern Europe (−0.623, *p* = 0.002) reveals a double burden: the region suffered both the baseline structural disadvantage and a disproportionately severe acute shock. The total estimated loss for the East was 1.74 years—56% greater than the 1.12-year loss in the West. This model successfully captures the central narrative of unequal suffering but remains a “black box,” identifying that the impact differed without specifying how or through what mechanism.

#### 3.1.5. Model 4 (Evaluating Vaccination as a Protective Moderator)

Model 4 examines whether the primary biomedical intervention, vaccination coverage, moderated the pandemic’s demographic impact. The results are clear: vaccination does not attain statistical significance as a direct predictor, and its inclusion degrades model fit (ΔAIC = +10.70). This critical finding does not imply biological inefficacy but rather suggests a misspecification of its role. At the macro, country-level of analysis, vaccination’s protective effect is likely not independent but is instead mediated—its benefit operates by reducing the ultimate outcome of interest, excess mortality. This model serves as a diagnostic, redirecting the analytical focus from specific interventions to broader mortality outcomes.

#### 3.1.6. Model 5 (Identifying the Proximate Mechanism: Excess Mortality)

Model 5 represents the analytical breakthrough. By introducing Excess mortality, the model substitutes the conceptual “pandemic period” with its quantifiable demographic consequence. The transformative improvement in fit (ΔAIC = −247.67) is unambiguous: excess mortality is the dominant proximate mechanism. Its highly significant coefficient (−0.085) quantifies the direct cost to life expectancy from the mortality shock. Consequently, the Pandemic year variable becomes statistically redundant, demonstrating that the crisis impacted longevity entirely through the channel of excess mortality. This re-specification correctly identifies the causal pathway, shifting the focus from the event itself to its measurable population-level outcome.

#### 3.1.7. Model 6 (The Final Parsimonious Model: Structure and Mechanism)

The final, optimized model (M6) achieves theoretical clarity by retaining only significant predictors. It synthesizes the findings: the secular trend (+0.124/year) persists; the East–West structural gap (−4.57 years) remains substantial; and excess mortality (−0.091/year per 1% increase) is the powerful proximate driver. Notably, it reveals that healthcare expenditure has a stronger independent protective effect than GDP, underscoring the importance of targeted health system investment over general economic wealth. This model provides a coherent, multi-layered explanation of European life expectancy dynamics, distinguishing between deep structural determinants, an immediate crisis mechanism, and the enduring progress of population health.

**Figure 7:**
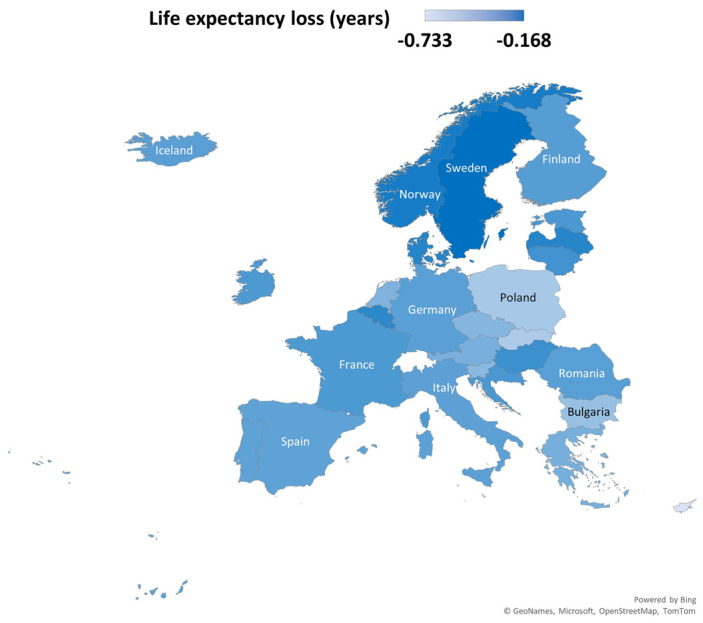
Country-specific estimated life expectancy loss attributable to pandemic excess mortality (2020–2023).

The spatial distribution of the pandemic’s demographic impact, derived from our multilevel model estimates, is presented in Figure 7. This map moves beyond tabular data to reveal the stark regional clustering of life expectancy loss, highlighting not only the severe burden borne by Central and Eastern Europe but also the notable resilience of the Nordic bloc, thereby providing a powerful visual synthesis of the crisis’s unequal continental footprint.

The application of our final model coefficient (Excess mortality, *β*_7_ = −0.091) to national excess mortality data reveals a nuanced and heterogeneous geography of demographic loss across Europe. Contrary to a simplistic East–West dichotomy, the most severe life expectancy reductions (exceeding 0.55 years) are concentrated in a Central European core (Slovakia, Poland, Czechia) and specific Southern European islands (Cyprus, Malta). This pattern suggests that structural vulnerabilities—such as healthcare systems in transition, high levels of comorbid disease burdens, and economies heavily reliant on specific sectors like tourism—were more predictive of severe demographic shocks than mere geographical longitude.

Notably, the Nordic countries (Sweden, Norway, Denmark) form a distinct resilience cluster, with losses contained between 0.17 and 0.27 years. This aligns with theories emphasizing the protective role of high institutional trust, social cohesion, and robust, equitable health systems. Significant intra-regional variations further challenge monolithic explanations: within Southern Europe, the severe impact on Cyprus and Malta contrasts with the moderate losses in Italy, Spain, and Portugal; within Central Europe, Hungary’s relatively contained loss (−0.33 years) deviates from the severe losses of its neighbors. These findings compel a move beyond regional generalizations towards a granular, country-specific understanding of health system resilience. The pandemic acted not as an equalizer but as a demographic stress test that magnified pre-existing, often mid-spectrum vulnerabilities, particularly in upper-middle-income economies with specific structural weaknesses, while saving both the most developed systems and some lower-income countries that may have experienced different epidemiological dynamics or implemented stringent early measures.

### 3.2. Residual Diagnostics and Model Validation

**Figure 8:**
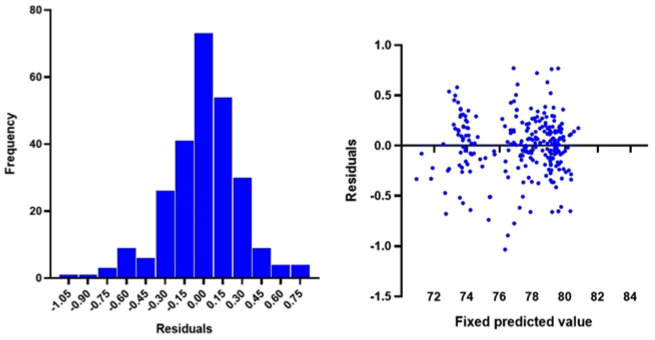
Residual diagnostics for the regression model.

The statistical robustness and inferential validity of the final multilevel model were rigorously assessed through a comprehensive suite of residual diagnostics. An examination of the distribution of the standardized conditional residuals, illustrated in Figure 8, indicates no substantive departure from normality.

The assumption of homoscedasticity—that the variance of the errors is constant across all levels of the predicted outcome—was evaluated via a scatterplot of the model’s fitted values against standardized residuals (Figure 8). The plot demonstrates a classic “null” pattern: the residuals are randomly and uniformly dispersed within a horizontal band centered around zero. Critically, there is no evidence of systematic patterning, such as fanning, curvature, or increasing/decreasing variance, which would indicate heteroscedasticity or model misspecification.

The Durbin-Watson statistic, calculated to assess autocorrelation within the longitudinal series for each country, yielded values proximate to 2, suggesting no significant temporal autocorrelation in the errors. Collectively, these diagnostic procedures affirm that the underlying assumptions of the linear mixed-effects model are tenable, thereby lending substantial credibility to the parameter estimates and associated hypothesis tests presented in the analysis.

## 4. Discussion

An estimated 131 million deaths from all causes occurred worldwide in 2020 and 2021 combined. Of these, approximately 15.9 million were attributed to the COVID-19 pandemic, based on excess mortality estimates that account for both deaths directly caused by SARS-CoV-2 infection and those indirectly resulting from related social, economic, and behavioral changes. Therefore, the significant effects of the COVID-19 pandemic across global populations have highlighted the importance of producing timely estimates to better understand this extraordinary event in relation to long-term population health trends [5].

The presented multilevel longitudinal analysis yields several principal findings that refine the understanding of the pandemic’s demographic impact. First, it quantified that the COVID-19 crisis reversed approximately five years of pre-pandemic progress in European life expectancy, with a model-adjusted net loss of 1.12 years. Second, it identified a profound and heterogeneous regional impact: while the loss in Western Europe was 1.12 years, Eastern Europe suffered a disproportionate loss of 1.74 years of life expectancy—a 56% more severe shock. The study’s core novelty lies in its mechanistic clarification and the consequent recalibration of what constitutes meaningful resilience. Our most significant finding is the identification of excess mortality as the complete mediator of the pandemic’s effect on life expectancy. Statistically, the binary pandemic period becomes non-significant once excess mortality is introduced into the model (β = −0.091, *p* < 0.001). This shifts the paradigm from analyzing a “pandemic impact” to analyzing a “mortality shock impact,” with direct implications for policy targeting and crisis monitoring. We acknowledge that the primacy of excess mortality over cause-specific mortality as a driver of life expectancy decline is well established in the literature [1,13,14,15]. Our contribution is not to rediscover this relationship but to quantify it precisely (β = −0.091) and to demonstrate how it renders the binary pandemic indicator redundant in a multilevel framework, thereby providing a replicable metric for future crisis assessment.

Furthermore, we disentangle the protective roles of structural factors, revealing that healthcare expenditure per capita exerts a stronger independent effect on life expectancy than GDP per capita. This underscores that it is not general wealth, but targeted investment in health system capacity, that builds demographic resilience. Conversely, and counterintuitively, national vaccination coverage did not emerge as a significant direct moderator in the final model. This suggests its primary benefit was mediated through reducing excess mortality, highlighting that biomedical interventions, while crucial, do not compensate for deep-seated structural vulnerabilities. Crucially, the non-significance of vaccination coverage in the final model does not indicate that vaccines were ineffective. Rather, it demonstrates that the protective effect of vaccination on life expectancy operates entirely through the reduction in excess mortality—a finding that is fully consistent with, and indeed confirms, high vaccine effectiveness at the population level. Readers should not interpret this result as evidence against vaccination.

A recent study from the Netherlands shows that a lower relative incidence of short-term deaths following COVID-19 vaccination suggests that vaccination is not associated with the observed excess mortality. Additionally, excess mortality during the COVID-19 pandemic at times exceeded the number of reported COVID-19-related deaths, also indicating that other factors may have contributed [21].

The percentage of the population receiving at least one vaccine dose served as a readily available and comparable metric across European countries during the study period. However, we acknowledge its crudeness: it does not capture the timing of vaccination, booster doses, waning immunity, or age-specific coverage patterns, all of which may influence population-level outcomes. This measure was chosen for its consistency across national reporting systems and its availability for the full panel, but future studies employing more granular indicators (e.g., time-updated coverage, age-standardized rates, or vaccine effectiveness metrics) may refine our understanding of the relationship.

Finally, the presented model partitions the East–West health gap, revealing that only about 21% of the 5.8-year disparity is explained by differences in GDP, health spending, and pandemic mortality. The remaining 79% represents a persistent, residual deficit, probably attributable to historical, economic, institutional, healthcare, lifestyle, and cultural factors. Decades of consistent gains in life expectancy across Europe began to slow around 2011, well before the onset of the COVID-19 pandemic. We observed that across all European regions, life expectancy showed a steady upward trend during the pre-pandemic period (2015–2019), with consistent annual improvements. This trajectory was abruptly disrupted in 2020, when the pandemic caused a sharp decline or, in some cases, stagnation. In 2021, life expectancy reached its lowest point in three regions, reflecting the delayed peak in mortality, with Eastern Europe experiencing the most pronounced drop—from 74.30 years in 2019 to 71.79 in 2021. Notably, by 2023, all regions except Eastern Europe had recovered to or even surpassed their pre-pandemic levels, indicating a demographic rebound (Figure 3).

A recent study, drawing on data and methods from the Global Burden of Diseases, Injuries, and Risk Factors Study 2021, examined trends in life expectancy at birth, causes of death, and exposure to risk factors across 16 European Economic Area countries between 1990 and 2021. The countries that sustained progress in life expectancy after 2011—namely Norway, Iceland, Belgium, Denmark, and Sweden—achieved this by continuing to reduce mortality from cardiovascular diseases and cancers. These improvements were supported by lower exposure to key risk factors, likely influenced by effective government policies. Notably, these same countries also maintained increases in life expectancy during 2019–2021, suggesting they were better equipped to cope with the COVID-19 pandemic [22]. Overall, the findings indicate that policies aimed at improving population health can also strengthen resilience to future public health crises. Thus, the pandemic did not create health inequality in Europe; it acted as a high-intensity spotlight, revealing and exacerbating the continent’s deepest and most stubborn structural fissures. The true novelty of this analysis is its multi-layered conclusion: sustainable demographic security requires long-term investment in equitable health systems, as acute crisis interventions, even when effective, cannot overcome foundational weaknesses.

In 2024, Pizzato et al. also analyzed 29 European countries over 4 years (2020–2023) to assess the impact of COVID-19 on total mortality, examining the associations between vaccination uptake, non-pharmaceutical interventions, and certain nation-level socioeconomic indices [23]. They estimated 1,642,586 excess deaths across all countries over the four years. Italy, Poland, and Germany were expected to have the highest number of excess fatalities during the 2020–2023 period, while Bulgaria, Lithuania, and Slovakia had the biggest excesses in relative terms. In Sweden, the age-standardized death rate was 1.8 per 10,000 people, while in Bulgaria, it was 24.7. An elevated excess death rate was substantially correlated with the proportion of the population living below the poverty line. On the other hand, the excess death rate was inversely correlated with GDP per capita, health expenditure, and the proportion of people who were vaccinated by the end of 2021 or 2022. They concluded that variations in socioeconomic settings and inadequate vaccination uptake in certain nations could be linked to the observed spatial disparities in overall mortality excess throughout Europe [23].

In a study by Islam et al., reductions in life expectancy in 2020 were observed for both men and women in 34 out of 37 upper-middle- and high-income countries or regions with reliable mortality data. The largest declines were recorded in Russia (men: −2.33 years; women: −2.14 years). Meanwhile, Bulgaria experienced the highest excess years of life lost per 100,000 population. Overall, more than 28 million excess years of life were lost across 31 countries in 2020, with men being more affected than women. The excess years of life lost due to the COVID-19 pandemic in 2020 were more than five times greater than those linked to the seasonal influenza epidemic in 2015 [13].

In 2023, Huang et al. conducted a study across 27 countries in Europe, Chile, and the USA, providing a comprehensive investigation of mortality variations during the COVID-19 period (2019–2023). They revealed varying mortality rates across countries during the COVID-19 pandemic. Although most of the 27 countries saw improvements in life expectancy after 2022, by May 2023, life expectancy in 22 of them had not yet fully returned to pre-pandemic levels. While the impact of COVID-19 remains more pronounced among older adults and men, particular attention should also be given to women aged 85 years and older [7].

The Global Burden of Diseases, Injuries, and Risk Factors Study (GBD) 2021 presents updated demographic estimates covering 204 countries and territories, as well as 811 additional subnational regions, spanning the period from 1950 to 2021. It placed particular focus on shifts in mortality and life expectancy during the COVID-19 pandemic, 2020–2021. Over the long term, global life expectancy at birth rose by 22.7 years, increasing from 49.0 years in 1950 to 71.7 years in 2021. However, between 2019 and 2021, life expectancy declined by 1.6 years, marking a reversal of historical progress. During this period, only 32 out of 204 countries and territories (15.7%) experienced an increase in life expectancy [5]. Our study on 29 European countries found similar results regarding the decline in life expectancy: the pandemic period was associated with an average reduction of 1.12 years in life expectancy after adjusting for pre-existing trends, with Eastern Europe suffering a disproportionate loss of 1.74 years. Life expectancy at birth is widely regarded as an indicator of a country’s social progress, the effectiveness of its health system, and the level of economic development. Therefore, all European countries must undertake targeted efforts to improve this measure by implementing economic and health policies that prioritize the well-being of their populations, as the authors already stated in a previous paper [24].

Although COVID-19 is no longer classified as a public health emergency since 5 May 2023, it continues to pose a major global health risk and remains a significant challenge for societies worldwide. Consequently, gaining a deeper understanding of the pandemic’s impact on morbidity and mortality, as well as the associated trends and changes over time, remains essential.

### 4.1. Limitations of the Study

First, the ecological study design, based on aggregated country-level data, precludes inferences about individual-level risk factors and may obscure important within-country inequalities, particularly across socio-economic groups. Second, while longitudinal, the observational nature of the data limits causal claims; despite robust modeling, unmeasured confounding factors may influence the observed associations. Third, the selection of variables, though theoretically grounded, is not exhaustive; factors such as health system governance quality, population health behaviors, and the stringency of non-pharmaceutical interventions were not included and could refine the model. Fourth, the nine-year timeframe, while capturing the pandemic shock, may be insufficient to assess the full long-term trajectory of recovery and potential scarring effects on life expectancy. Furthermore, while our model treats excess mortality as a predictor of life expectancy, we acknowledge the conceptual overlap between these two indicators, as excess mortality is a component of the mortality schedules from which life expectancy is derived. An alternative analytical specification, wherein excess mortality serves as the dependent variable regressed on structural factors, represents a complementary approach that may directly assess the determinants of pandemic vulnerability.

While the positive bivariate correlation between excess mortality and vaccination coverage (r = 0.497) is most parsimoniously explained by temporal confounding—countries implemented mass vaccination campaigns precisely during periods of highest pandemic intensity—we acknowledge that other explanations are theoretically possible. These may include, for example, unmeasured differences in health-seeking behavior, testing capacity, or mortality reporting standards across countries and over time. Our interpretation of this correlation as primarily temporal in nature is supported by the fact that it disappears in multivariate models controlling for time and regional structure, but we caution readers against drawing causal inferences from bivariate associations.

Finally, the reliance on official excess mortality data, though the gold standard, may still be subject to cross-national differences in death registration completeness and timeliness, particularly during crisis periods.

### 4.2. Prospects for Future Research

Future investigations should utilize individual-level longitudinal data to disentangle the pandemic’s heterogeneous impact across socio-economic, occupational, and ethnic groups, moving beyond national aggregates to the micro-foundations of health inequality. Furthermore, research must extend the temporal horizon to examine the long-term scarring effects of the crisis on mortality trends and cohort life expectancy, determining whether the observed losses represent a permanent setback or a temporary shock.

An alternative analytical specification—one that treats excess mortality as the dependent variable regressed on structural factors such as healthcare expenditure, GDP, and vaccination coverage—would address a complementary research question concerning the determinants of the mortality shock itself. While beyond the scope of the present investigation, which focuses on life expectancy as the ultimate demographic outcome, future studies would benefit from this parallel approach.

Finally, a critical avenue involves causal inference studies designed to evaluate the efficacy of specific policy interventions—such as healthcare financing reforms, primary care strengthening, or targeted prevention programs—in mitigating excess mortality during systemic health crises, thereby translating descriptive findings into actionable policy evidence.

## 5. Conclusions

This study highlights that the COVID-19 pandemic inflicted a severe and regressive demographic shock on Europe, erasing years of progress in life expectancy and acting as a powerful magnifier of pre-existing inequalities. The disproportionate burden borne by Eastern Europe reveals that the crisis translated into a significantly larger mortality shock in regions with weaker structural foundations. Crucially, our findings shift the focus from the pandemic as an event to excess mortality as the key measurable outcome, underscoring that resilient health systems, proxied by higher per capita expenditure, offer greater protection than general economic wealth alone. Vaccination remains a critical public health tool, and our results are a statistical artifact of mediation, not a reflection of biological reality.

Consequently, the policy imperative extends beyond pandemic preparedness to address the deep-seated, multi-faceted determinants of the persistent East–West health gap, which remains largely unexplained by conventional economic indicators. The legacy of this crisis should therefore catalyze a commitment to long-term, structural investments in health system capacity and equity, as reactive interventions, however technologically advanced, cannot compensate for foundational vulnerabilities. Ultimately, securing population health and demographic resilience requires building systems that are robust not only to acute shocks but to the chronic stresses of inequality and underinvestment.

## Figures and Tables

**Table 1:** Descriptive statistics of the analytical sample.

Variable	N	Mean	Standard Deviation	Min	Max
Life expectancy (years)	261	77.37	3.72	68.0	81.8
Gross domestic product PPSs *	261	33.495	14.49	13.587	93.489
Healthcare expenditure PPSs *	261	2.787	1.134	797	5.507
Percentage of population vaccinated with a single dose	261	21.48%	31.78%	0%	94.28%
Population aged 65+	261	19.12%	2.62%	12.50%	24.22%
Population density	261	171.5	283.6	3.3	1.727.3
Excess mortality	261	+4.59%	7.23%	−6.64%	+38.01%

Note: * purchasing power standards.

**Table 2:** Comparative descriptive statistics: pre-pandemic (2015–2019) vs. pandemic period (2020–2023).

Variable	Pre-COVID-19 (*N* = 145)		COVID-19 Period (*N* = 116)		Difference	Sig. (*p*)
	Mean	Standard Deviation	Mean	Standard Deviation		
Life expectancy	77.40	3.58	77.32	3.90	−0.08	0.869
Gross domestic product PPSs *	30,719.31	12,871.81	36,964.12	15,665.40	6244.81	<0.001
Healthcare expenditures PPSs *	2503.51	1046.13	3142.14	1144.90	638.62	<0.001
COVID-19 vaccination with one dose (%)	0.00	0.00	48.3%	31.2%	48.3%	<0.001
Excess mortality (%)	0.00	0.00	10.3%	7.65	10.3%	<0.001

Note: * purchasing power standards.

**Table 3:** Regional disaggregation of life expectancy.

Region	Pandemic Year	Mean	N *	Standard Deviation	Difference
North	Pre-COVID-19	77.26	45	4.39	0.28
	COVID-19 period	77.55	36	4.57	
South	Pre-COVID-19	79.22	35	2.02	−0.11
	COVID-19 period	79.11	28	2.17	
East	Pre-COVID-19	73.95	35	2.34	−0.60
	COVID-19 period	73.36	28	2.89	
West	Pre-COVID-19	79.50	30	0.60	0.03
	COVID-19 period	79.53	24	0.78	

Note: * country-year observations for each region.

**Table 4:** Pre-pandemic regional baselines: structural determinants of population health.

Variable	East	North	South	West	Total
Average life expectancy	73.69	77.39	79.17	79.51	77.37
Average gross domestic product PPSs *	23,712.44	38,271.08	26,922.84	45,410.33	33,494.78
Average healthcare expenditures PPSs *	1784.23	3102.53	2266.82	4092.16	2787.35
Average % of population vaccinated with a single dose	15.49	22.95	24.00	23.33	21.48
Average excess mortality (%)	5.55	3.42	5.42	4.25	4.59

Note: * purchasing power standards.

**Table 5:** Bivariate correlations among key study variables.

Pearson Correlation	Life Expectancy	GDP PPS	Healthcare Expenditures PPS	Percentage of Population Vaccinated with a Single Dose	Excess Mortality
Life expectancy	1.000				
Gross domestic product PPSs	0.560 **	1.000			
Healthcare expenditures PPSs	0.705 **	0.737 **	1.000		
Percentage of population vaccinated with a single dose	0.136 *	0.322 **	0.367 **	1.000	
Excess mortality	−0.104	0.074	0.127 *	0.497 **	1.000

Note: ** correlation is significant at the 0.01 level (2-tailed); * correlation is significant at the 0.05 level (2-tailed). Background colors are used to enhance the visibility of the displayed results.

**Table 6:** Staged multilevel model building.

Model	Name and Specification	Theoretical Rationale/Research Question Tested	Key Result and Interpretation
M_0_	Null (Empty) Model *Life_expectancy_it_ = β*_0_* + u_i_ + ε_it_*	Partition total variance into within-country and between-country components. Establish baseline ICC.	*ICC = Between-country variance/Total variance = 13.84/14.14 = 97.87%.* The majority of variance lies *between* countries, strongly justifying the multilevel approach in our study.
M_1_	Time + Pandemic Effect *Life_expectancy_it_ = β*_0_ *+ β*_1_ *× Year_t_ + β*_2_ *× Pandemic_year_t_ + u_i_ + ε_it_*	Quantify the net average effect of the pandemic period, controlling for the pre-existing secular trend.	*Pandemic_year_t_ β*_2_ = −1.223 (*p* < 0.001). The pandemic period is associated with a net loss of 1.22 years, erasing ~5 years of pre-pandemic progress.
M_2_	+ Regional Structure *Life_expectancy_it_ = β*_0_ *+ β*_1_ *× Year_t_ + β*_2_ *× Pandemic_year_t_ + β*_3_ *× Region_i_ + u_i_ + ε_it_*	Account for fixed structural differences between Europe’s major geographic regions. Does region explain baseline disparities?	*Region_i_* [East] *β*_3_ = −5.826 (*p* = 0.003). Confirms a massive pre-pandemic East–West gap of 5.83 years. Pandemic effect unchanged.
M_3_	+ Differential Impact (Interaction) *Life_expectancy_it_ = β*_0_ *+ β*_1_ *× Year_t_ + β*_2_ *× Pandemic_year_t_ + β*_3_ *× Region_i_ + β*_4_*× (Pandemic_year_t_ × Region_i_) + u_i_ + ε_it_*	Test for heterogeneity in pandemic impact: Was the shock uniform, or did it vary by region?	*Pandemic_year_t_ × Region_i_* [East] *β*_4_ = −0.623 (*p* = 0.002). Impact was 56% larger in East (−1.74 yrs) than in West (−1.12 yrs). Key finding of disparity.
M_4_	+ Vaccination as Moderator *Life_expectancy_it_ = β*_0_ *+ β*_1_ *× Year_t_ + β*_2_ *× Pandemic_year_t_ + β*_3_ *× Region_i_ + β*_4_*× (Pandemic_year_t_ × Region_i_) + β*_5_ *× Vaccination_it_ + β*_6_*× (Pandemic_year_t_ × Vaccination_it_) + u_i_ + ε_it_*	Test if vaccination coverage moderated the pandemic’s impact on life expectancy.	*Vaccination_it_ β*_5_ = n.s. (*p* = 0.964). Interaction redundant. No direct, independent moderating effect found at the macro level.
M_5_	Mechanism: Excess Mortality *Life_expectancy_it_ = β*_0_ *+ β*_1_ *× Year_t_ + β*_2_ *× Pandemic_year_t_ + β*_3_ *× Region_i_ + β*_4_*× (Pandemic_year_t_ × Region_i_) + β*_7_ *× Excess_mortality_it_ + u_i_ + ε_it_*	Introduce the proximate mechanism: does excess mortality directly drive life expectancy loss, subsuming the pandemic indicator?	*Excess_mortality_it_ β*_7_ = −0.085 (*p* < 0.001). *Pandemic_year_t_* becomes n.s. (*p* = 0.527). Breakthrough: *The entire pandemic effect is mediated by excess mortality*. East interaction remains *β*_3_ *= −5.549* (*p* = 0.004)
M_6_	+ Structural Economic Controls *Life_expectancy_it_ = β*_0_ *+ β*_1_ *× Year_t_* + *β*_3_ *× Region_i_ + β*_7_ *× Excess_mortality_it_ + β*_8_ *× GDP_PPS_it_ + β*_9_ *× Healthcare_expenditures_PPS_it_ + u_i_ + ε_it_*	Optimized Parsimonious Model. Remove non-significant pandemic terms. Test if structural resources (GDP, Healthcare expenditures) have independent protective effects *after* controlling for the mortality shock.	*Excess_mortality_it_ β*_7_ = −0.091 (*p* < 0.001) *Healthcare_expenditures_PPS_it_β*_9_ = +0.000327 (*p* = 0.049). *GDP_PPS_it_ β*_8_ = +1.74 × 10^−5^ (*p* = 0.020). *Region_i_* [East] *β*_3_ = −4.574 (*p* = 0.009).

## Data Availability

The original contributions presented in this study are included in the article. Further inquiries can be directed to the corresponding author(s).
